# Comparison of mRNA-1273 and BNT162b2 SARS-CoV-2 mRNA Vaccine Immunogenicity in Kidney Transplant Recipients

**DOI:** 10.3389/ti.2021.10026

**Published:** 2022-01-04

**Authors:** Maria C. Haller, Robert A. Kaiser, Simon Langthaler, Clara Brandstetter, Petra Apfalter, Heidrun Kerschner, Daniel Cejka

**Affiliations:** ^1^ Internal Medicine III—Nephrology, Transplantation Medicine, Rheumatology, Ordensklinikum, Linz, Austria; ^2^ Section for Clinical Biometrics, Center for Medical Statistics, Informatics and Intelligent Systems, Medical University of Vienna, Vienna, Austria; ^3^ Institute for Hygiene, Microbiology and Tropical Medicine, Ordensklinikum, Linz, Austria

**Keywords:** Covid-19, Sars-CoV-2, kidney transplantation, vaccination, mRNA vaccine

Dear Editors,

Kidney transplant recipients are at high risk for severe COVID-19 disease or death in case of SARS-CoV-2 infection (1). There is growing evidence suggesting that anti-SARS-Cov2-antibody response is markedly blunted in kidney transplant patients after vaccination (2). Severe COVID- 19 and COVID-19-related death has been recently reported in kidney transplant recipients despite prior complete (two dose) vaccination with SARS-CoV-2 mRNA vaccines (3). (4).

In this retrospective cohort study involving 320 prevalent kidney transplant recipients from a single transplant center (Ordensklinikum Linz—Elisabethinen hospital), anti- Spike (S) protein IgG antibody titers were measured 3–6 weeks [BNT162b2: median 28 days (IQR: 6 days), mRNA1273: median 28 days (IQR: 8 days)] after administration of the second dose of either mRNA-1273 or BNT162b2 SARS-CoV-2 vaccine. Vaccinations took place between January 15th and June 8th, 2021 according to the Austrian national SARS-CoV-2 vaccination program. Vaccine doses were allocated by the Austrian government depending on availability. Patients were vaccinated ranked by age beginning with the oldest as soon as vaccines were available. Allocation to a certain vaccine (BNT162b2 or mRNA-1273) was therefore determined by the vaccination progress in our kidney transplant cohort and vaccine availability at that time. A two-dose vaccination regimen was applied, with 3-weeks (BNT162b2) and 4-weeks (mRNA-1273) intervals between the first and second vaccination, regardless of a history of prior infection with SARS-CoV-2.

Anti-SARS-CoV-2-antibodies directed against the receptor binding domain (RBD) of the S1 subunit of the Spike (S) protein were measured with the SARS-CoV-2 IgG II Quant assay (Abbott Ireland Diagnostics Division, Finisklin Business Park, Sligo, Ireland), which was reported to have high sensitivity and specificity for detection of anti-SARS-CoV-2 S-protein antibodies (5) and high correlations with anti-SARS-CoV-2 neutralizing antibodies (6). Results were reported in BAU/ml (binding antibody units). Differences between vaccine groups (mRNA-1273 vs BNT162b2) in S-antibody-positivity were tested for statistical significance using the Chi^2^-Test. To further investigate the impact of vaccine type on S-antibody-positivity, we computed a multivariate logistic regression model taking potential confounding factors of seroconversion after SARS-COV2 vaccination into account. Results are reported as odds ratios (OR) and 95% confidence intervals (95% CI). We performed a complete case analysis as covariate information was missing in one patient only for the multivariate model. The study was approved by the Ethics Committee of the Johannes Kepler University Linz (ID: 1100/2021). Patients provided written informed consent. Patient demographics and additional analyses are shown in the Supplementary Material.

Anti-S-antibody positivity was detected in 51% of the patients in our study cohort. A higher proportion of mRNA-1273 vaccinated patients achieved antibody-positivity compared to those vaccinated with BNT162b2 (61.6 vs 47.7%, *p* = 0.037, Chi^2^-test). After correction for age, diabetes status, sex, serum albumin and serum creatinine, the odds ratio for anti-S- antibody seroconversion was significantly higher for mRNA-1273 vaccinated patients compared to BNT162b2 in a multivariate regression analysis (odds ratio: 2.12, 95% confidence interval: 1.16 to 3.87, *p* = 0.013, Figure 1). After exclusion of patients with a history of prior SARS-CoV-2-infection [*N* = 21; 17 patients with BNT162b2, four patients with mRNA-1273; six patients with IgG antibodies directed against the nucleocapsid (N) protein, 15 Patients with positive SARS-CoV-2 polymerase chain reaction (PCR) test], results remained similar. In patients without prior SARS-CoV-2 infection (*N* = 299) a higher proportion of patients vaccinated with mRNA-1273 achieved seropositivity compared to patients vaccinated with BNT162b2 (59.4 vs 44.3%, *p* = 0.027, Chi^2^-test). The odds ratio for seroconversion was higher in mRNA-1273-vaccinated patients compared to BNT162b2-vaccinated patients in multivariate analysis (OR: 2.2, 95% CI: 1.19 to 4.08, *p* = 0.011).

**FIGURE 1:**
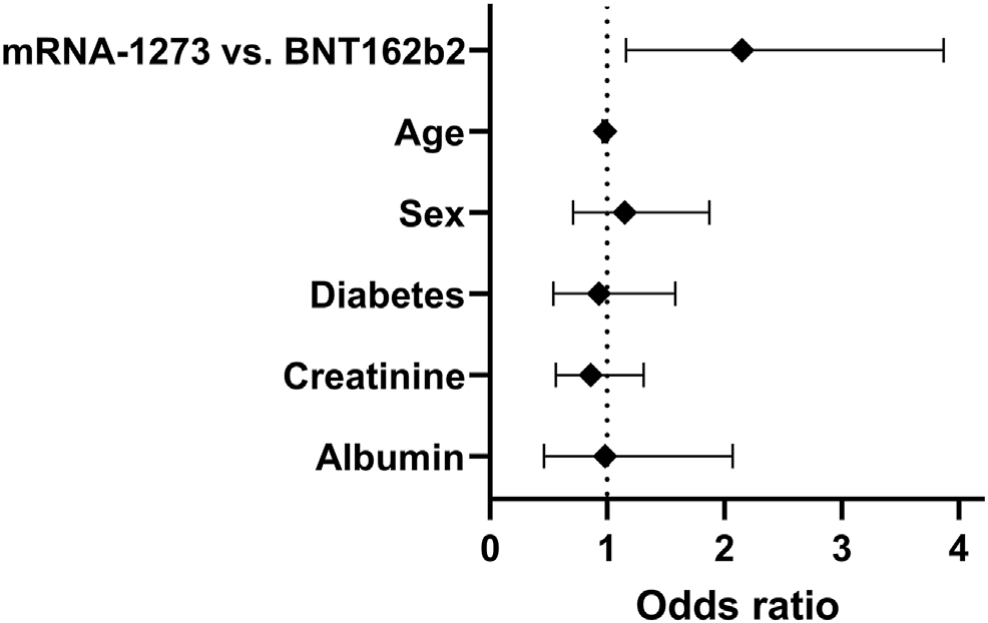
Odds ratios and 95% confidence intervals of a multivariate logistic regression analysis for anti-S-antibody seroconversion after 2 doses of SARS-CoV-2 mRNA vaccine in 320 prevalent kidney transplant patients irrespective of prior history of SARS-CoV-2 infection.

Reasons for a higher rate of seroconversion after mRNA-1273 vaccination compared to BNT162b2 are currently uncertain. Possible explanations include differences in mRNA content per vaccine dose, differences in mRNA modification or differences in the lipid formulation between the vaccines, all of which may influence expression of spike (S)-proteins and therefore immunogenicity. A limitation of this study is the lack of data in cellular immune responses, which may underestimate the immunogenicity of the vaccines. Another limitation is the retrospective nature of this study. However, similar results were recently reported in another observational study on immunogenicity of the mRNA-1273 and BNT162b2 vaccines in patients on renal replacement therapy (7), corroborating our findings.

In conclusion, vaccination with mRNA-1273 is associated with higher odds of anti-S-antibody seroconversion compared to vaccination with BNT162b2 in prevalent kidney transplant recipients.

## Data Availability

The raw data supporting the conclusions of this article will be made available by the authors, without undue reservation.
