# Supplementary material for: Comparison of mRNA-1273 and BNT162b2 SARS-CoV-2 mRNA Vaccine Immunogenicity in Kidney Transplant Recipients
Source: Transpl Int. 2022 Jan 4;35:10026. doi: 10.3389/ti.2021.10026 (PMC8842265; doi:10.3389/ti.2021.10026)
Supplement: Supplementary file 1 [file datasheet1.docx]

Supplementary material

# Supplementary Patients and Methods

Patient characteristics and laboratory data were extracted by chart review. History of SARS-CoV-2 infection was defined as either seropositivity for anti-N-antibodies at the time of anti-S-antibody titer control (to detect previous asymptomatic SARS-Cov2-infection), or a history of a positive SARS-CoV-2 polymerase chain reaction (PCR) test result, which was obtained upon clinical suspicion of COVID-19, occurring before the 1^st^ vaccine dose irrespective of the severity of clinical symptoms. Routine screening of patients for asymptomatic SARS-CoV-2 infection was not performed.

Data were analyzed retrospectively using SAS (SAS Institute, Car, USA, Version 9.4) and plotted using GraphPad Prism (GraphPad Software, San Diego, USA) software. Differences between vaccine groups (mRNA-1273 vs. BNT162b2) in S-antibody-positivity were tested for statistical significance using the Chi²-Test. To further investigate the impact of vaccine type on S-antibody-positivity, we computed a For the multivariate logistic regression model potential confounding factors for seroconversion after SARS-COV-2 vaccination were included: age (linear), diabetes status (yes/no), sex (male/female), serum albumin (linear) and serum creatinine (linear)A sensitivity analysis was conducted by excluding patients with prior SARS-CoV2-infection as this infection might have served as priming event causing higher anti-S-antibody titers after vaccination, which has been shown at least for immunocompetent individuals^S1^.

# Supplementary Table

**Table S1**

Patient characteristics.

BMI…body mass index; ADPKD…autosomal dominant polycystic kidney disease; mTORi…mammalian target of rapamycin inhibitor

# Supplementary Figure


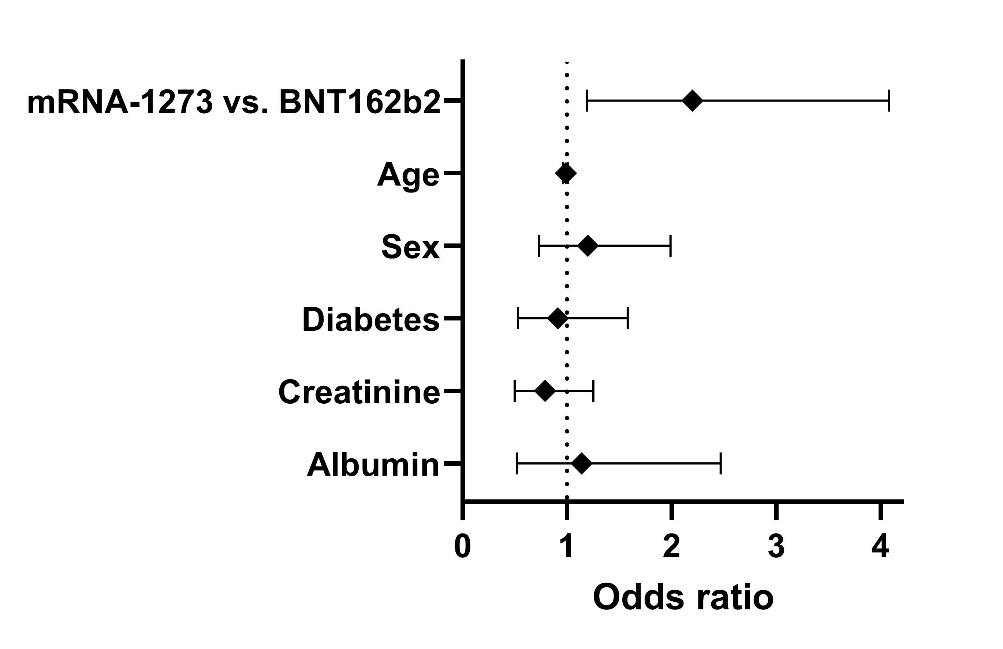


**Figure S1**

Odds ratios and 95% confidence intervals of a multivariate logistic regression analysis for anti-S-antibody seroconversion after 2 doses of SARS-CoV-2 mRNA vaccine in 299 prevalent kidney transplant patients without a history of SARS-CoV-2 infection.

# Authors’ Contributions

Research idea and study design: MCH, RAK, PA, HK, DC
Data acquisition: RAK, SL, HK, CB, DC
Data analysis/interpretation: MCH, RAK, SL, PA, HK, CB, DC
Statistical analysis: MCH, DC
Supervision or mentorship: DC
Each author contributed important intellectual content during manuscript drafting or revision and agrees to be personally accountable for the individual’s own contributions and to ensure that questions pertaining to the accuracy or integrity of any portion of the work, even one in which the author was not directly involved, are appropriately investigated and resolved, including with documentation in the literature if appropriate.

# Supplementary References

**S1.** Ebinger JE, Fert-Bober J, Printsev I, et al. Antibody responses to the BNT162b2 mRNA vaccine in individuals previously infected with SARS-CoV-2. *Nat Med.* 2021;27(6): 981-984.
